# Overexpression of SrUGT76G1 in Stevia alters major steviol glycosides composition towards improved quality

**DOI:** 10.1111/pbi.13035

**Published:** 2018-12-19

**Authors:** Mi Jung Kim, Junshi Zheng, Ming Hui Liao, In‐Cheol Jang

**Affiliations:** ^1^ Temasek Life Sciences Laboratory National University of Singapore Singapore Singapore; ^2^ Department of Biological Sciences National University of Singapore Singapore Singapore

**Keywords:** SrUGT76G1, Stevia, steviol glycosides, Reb A/stevioside ratio, Reb C/dulcoside A ratio

## Abstract

Steviol glycosides (SGs) are extracted from Stevia leaves for use as a natural sweetener. Among SGs, stevioside is most abundant in leaf extracts followed by rebaudioside A (Reb A). However, Reb A is of particular interest because of its sweeter and more pleasant taste compared to stevioside. Therefore, the development of new Stevia varieties with a higher Reb A to stevioside ratio would be desirable for the production of higher quality natural sweeteners. Here, we generated transgenic Stevia plants overexpressing Stevia *UDP‐glycosyltransferase 76G1* (*SrUGT76G1*) that is known to convert stevioside to Reb A through 1,3‐β‐d‐glucosylation *in vitro*. Interestingly, by overexpressing *SrUGT76G1*, the Reb A to stevioside ratio was drastically increased from 0.30 in wild‐type (WT) plants up to 1.55 in transgenic lines without any significant changes in total SGs content. This was contributed by a concurrent increase in Reb A content and a decrease in stevioside content. Additionally, we were able to find an increase in the Reb C to dulcoside A ratio in transgenic lines. Using the glutathione S‐transferase‐tagged SrUGT76G1 recombinant protein for an *in vitro* glucosyltransferase assay, we further demonstrated that Reb C can be produced from the glucosylation of dulcoside A by SrUGT76G1. Transgenic Stevia plants having higher Reb A to stevioside ratio were visually indistinguishable from WT plants. Taken together, our results demonstrate that the overexpression of *SrUGT76G1* in Stevia is an effective way to generate new Stevia varieties with higher proportion of the more preferred Reb A without compromising on plant development.

## Introduction

*Stevia rebaudiana* Bertoni is unique in its ability to produce steviol glycosides (SGs). As these SGs are generally 150–300 times sweeter than sucrose, they can be extracted for use as a nonnutritive sweetener (Lemus‐Mondaca *et al*., 2012). The leaves of Stevia accumulate the highest SGs content composing 4%–20% of the dry weight but the actual abundance of SGs differs between cultivars (Lemus‐Mondaca *et al*., 2012). In general, stevioside is the predominant SG present followed by rebaudioside A (Reb A) and Reb C. Dulcoside A, Reb F, steviolbioside, Reb D and Reb E are also frequently detected. By also taking into account the SGs that are only found in trace amounts from certain cultivars of Stevia, a total of more than 30 SGs are currently known to be produced in Stevia (Ceunen and Geuns, 2013).

SGs are synthesized from the glycosylation of steviol aglycone, which is derived from the methylerythritol phosphate (MEP) pathway. Each SGs has different number and combination of sugar moieties attached at the C_19_ or the C_13_ position of steviol (Ceunen and Geuns, 2013). All SGs contain β‐d‐glucose as their common sugar moiety but some SGs such as Reb C, Reb F and dulcoside A also have rhamnose and xylose added along with glucose. The addition of the activated sugars to aglycone acceptors is carried out by UDP‐glycosyltransferase (UGTs) (Richman *et al*., 2005). UGTs are considered to be promiscuous but they exhibit regioselectivity in the substrates they convert (Hansen *et al*., 2003). For the biosynthesis of SGs, four UGTs, SrUGT74G1, SrUGT76G1, SrUGT85C2 and SrUGT91D2, have been identified in Stevia so far. These Stevia UGTs contain the highly conserved plant secondary product glycosyltransferase (PSPG) motif of plant‐derived family 1 UGTs on their C‐terminus (Gachon *et al*., 2005; Richman *et al*., 2005). Each of them catalyses the addition of a sugar moiety at specific positions. SrUGT85C2 and SrUGT74G1 are known to glucosylate the C_13_ hydroxyl position and the C_19_ carboxylic acid position of the steviol aglycone respectively (Richman *et al*., 2005). On the other hand, SrUGT91D2 is able to further glucosylate the glucose attached on either C_13_ or C_19_ position to form a 1,2‐β‐d‐glucosidic linkage (1,2‐β‐d‐glucosylation) in the absence of a 1,3‐glucose (Olsson *et al*., 2016). For SrUGT76G1, it catalyses the glucosylation of the glucose moieties as well but forms a 1,3‐β‐d‐glucosidic linkage (1,3‐β‐d‐glucosylation) instead, and the presence of a 1,2‐glucose at SGs does not affect its activity (Olsson *et al*., 2016; Richman *et al*., 2005). The most commonly suggested route of synthesis for Reb A in Stevia involves an initial glucosylation by SrUGT85C2 on the steviol aglycone to form steviolmonoside (Figure S1; Humphrey *et al*., 2006; Richman *et al*., 2005). This might be followed by the addition of another glucose moiety by SrUGT91D2 to produce steviolbioside (Figure S1; Olsson *et al*., 2016). Next, SrUGT74G1 glucosylates steviolbioside to stevioside, and SrUGT76G1 then adds the final glucose required to give Reb A (Figure S1; Humphrey *et al*., 2006; Richman *et al*., 2005).

Although SGs are generally sweet, organoleptic properties of individual SGs depend on the combination of sugar moieties attached to steviol (Hellfritsch *et al*., 2012). Therefore, other than increasing overall SGs content, there is also a preference for Stevia varieties that can produce the more pleasant tasting SGs in greater proportions. Comparing between the two most abundant SGs in Stevia, Reb A is sweeter and less bitter tasting than stevioside and is thus more valuable as a sweetener (Singla and Jaitak, 2016). In the SGs biosynthesis pathway, stevioside can be converted to Reb A by SrUGT76G1 (Richman *et al*., 2005). Furthermore, SrUGT76G1 is also involved in the biosynthesis of Reb M, which has a more superior taste profile than Reb A but has only been detected in trace amounts in certain Stevia cultivars (Olsson *et al*., 2016; Prakash *et al*., 2014).

For increasing the levels of specific glycosylated metabolites, overexpression of the UGTs involved has been shown to be a feasible approach in plants. In *Rhodiola sachalinensis*, which is well‐known for the production of salidroside, the overexpression of *RsUGT73B6* led to an increase in salidroside content (Ma *et al*., 2007). Additionally, overexpression of *AtUGT73C6* and *AtUGT71C5* in Arabidopsis has also been demonstrated to increase brassinosteroid glucoside and abscisic acid‐glucose ester respectively (Husar *et al*., 2011; Liu *et al*., 2015). Therefore, the overexpression of Stevia *UGTs* in Stevia may increase total SGs content or promote the synthesis of preferred SGs.

Previously, we described a method for the *Agrobacterium*‐mediated transformation of Stevia and successfully increased the SGs content in the leaves through the overexpression of two pathway genes, *SrDXS1* and *SrKAH* (Zheng *et al*., 2018). Here, we generated transgenic Stevia plants overexpressing *SrUGT76G1*. Interestingly, instead of increasing the total SGs content, we found a significant improvement in the Reb A to stevioside ratio from 0.30 in the control to 1.55 in the *SrUGT76G1‐*overexpression lines without any negative effects on growth and development. Moreover, we observed an increase in the Reb C content relative to dulcoside A. Analysis of *in vitro* assay confirmed that in addition to Reb A conversion from stevioside, SrUGT76G1 could catalyse 1,3‐β‐d‐glucosylation on C_13_‐positioned glucose of dulcoside A to produce Reb C as well.

## Results

### Transgenic Stevia plants overexpressing *SrUGT76G1*

**Figure 1:**
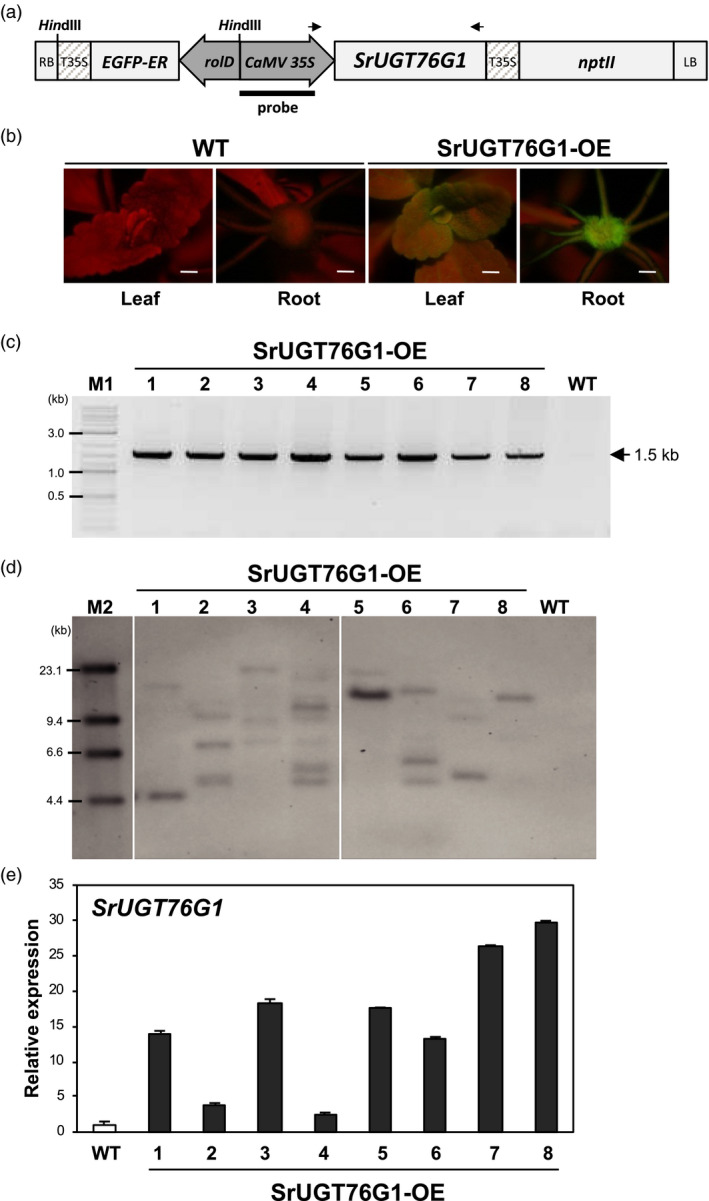
Molecular analysis of transgenic Stevia plants. (a) Schematic representation of T‐DNA region of the Stevia transformation construct (pK7WG2D‐SrUGT76G1). RB and LB, right and left border; rolD, rol root loci D promoter; EGFP‐ER, enhanced green fluorescent protein gene fused to endoplasmic reticulum targeting signal; T35S and CaMV35S, terminator and promoter of cauliflower mosaic virus 35S gene; *nptII*, neomycin phosphotransferase marker gene; *Hin*dIII, Enzyme site used for Southern blot analysis. Arrows indicate primers for genomic DNA PCR. (b) Images of GFP signal from leaves and roots of SrUGT76G1‐OE lines under a fluorescence stereomicroscope. WT, wild‐type. Scale bar = 1 mm. (c) Genomic DNA (gDNA) PCR amplification of SrUGT76G1 from the gDNAs of each transgenic line using forward and reverse primers specific to 35S promoter region and to the 3′ end of SrUGT76G1 respectively. (d) Southern blot analysis showing transgene copy number. gDNAs from each line were digested with *Hin*dIII and probed with DIG‐labelled probe specific for full length of CaMV 35S promoter. Only one individual from each line was cropped out from the original blot in Figure S2 and shown here. (e) Transcript levels of *SrUGT76G1* in SrUGT76G1‐OE lines. The relative fold change in *SrUGT76G1* expression level among the transgenic lines were normalized to that of WT and expressed as mean ± SE (*n *=* *3). M1; 2‐Log DNA ladder, M2; DIG‐labelled DNA Molecular Weight Marker II‐Lambda *Hin*dIII‐digested marker.

Since SrUGT76G1 has been known to convert stevioside to Reb A, steviobioside to Reb B, and Reb D to Reb M (Olsson *et al*., 2016; Richman *et al*., 2005), we hypothesize that its overexpression in Stevia could increase or alter the proportion of these SGs. Therefore, we cloned the full‐length open reading frame (ORF) of *SrUGT76G1* into pK7WG2D under the control of the cauliflower mosaic virus (CaMV 35S) promoter for the *Agrobacterium*‐mediated transformation of Stevia (Figure 1a). Using our previously established transformation method of Stevia that employs green fluorescent protein (GFP) as a visual marker (Zheng *et al*., 2018), we were able to generate eight transgenic lines emitting GFP signals (Figure 1b). To avoid affecting the normal functions of SrUGT76G1, no tag was fused to the protein.

To verify the integration of the exogenous *SrUGT76G1* in transgenic Stevia, we carried out a genomic PCR analysis with the genomic DNA extracted from each *SrUGT76G1*‐overexpression lines (SrUGT76G1‐OE). A band corresponding to the expected size of the *SrUGT76G1* transgene was detected in all the transgenic lines except WT (Figure 1c and Figure S2). For further investigation into the copies of transgene present in each line, we then performed a digoxigenin (DIG)‐based Southern blot analysis with *Hin*dIII‐digested genomic DNA extracted from each line using a CaMV 35S promoter‐specific probe. Figure 1d shows that only line #8 contained a single copy of transgene while other lines had two or more copies of the transgene integrated.

Next, we analysed the transcript levels of *SrUGT76G1* in the SrUGT76G1‐OE lines using qRT‐PCR. Figure 1e shows that the transcript levels of *SrUGT76G1* were approximately 3‐to‐30‐folds higher in the SrUGT76G1‐OE lines as compared to WT, with lines #8 and #4 being the highest and lowest expressors respectively. When compared to the copies of transgene present, more copies of transgene did not correlate with higher expression levels in the overexpression lines. This lack of correlation has long been appreciated in transgenesis (Hobbs *et al*., 1993). It is suggested to be caused by epigenetic silencing due to the surrounding chromatin environment, also known as position effects, and the interactions between homologous sequences, also known as homology‐dependent gene silencing (Kohli *et al*., 2003; Matzke and Matzke, 1998; Rajeevkumar *et al*., 2015). For further analysis on the effect of SGs abundance and/or changes in SGs ratio, we selected two lines with the highest expression of SrUGT76G1, which are #7 and #8. Lines #1 and #5 were also selected as they seemed to have low copies of the transgene inserted and showed relatively high levels of SrUGT76G1 expression. We also included line #2 as an internal control line that shows only threefold higher expression of *SrUGT76G1*.

### Alteration of steviol glycosides composition in transgenic Stevia plants

**Figure 2:**
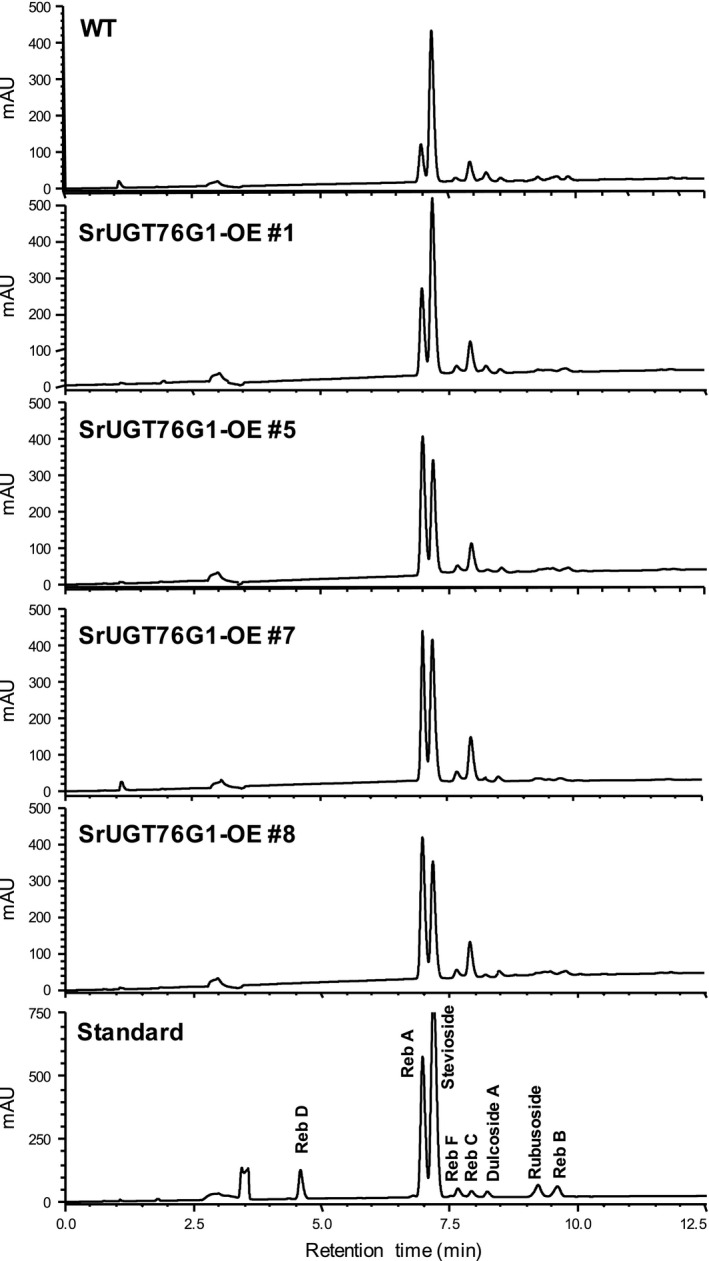
HPLC chromatogram showing steviol glycosides (SGs) content from four SrUGT76G1‐OE lines. Individual SGs are identified by their alignment with the retention time of authentic standards. WT, wild‐type; mAU, milli‐Absorbance Units.

To measure the SGs content in SrUGT76G1‐OE lines, we multiplied the plants through *in vitro* cutting propagation and harvested leaves from the same nodal position after hardening in the soil. Extracted SGs from the dried leaves were analysed using high‐performance liquid chromatography (HPLC) and individual SGs were identified by the alignment of their retention time with that of authentic standards (Figure 2). The SGs content in the WT and the internal control, line #2, were comparable (Figure S3a). Intriguingly, by comparing the representative chromatograms of each transgenic line to that of WT, we found a noticeable change in the relative abundance of Reb A to stevioside (Figure 2). In all SrUGT76G1‐OE lines except #1, the Reb A peak even surpassed the peak for stevioside (Figure 2). However, we could not detect any additional SGs that were previously mentioned to be products of *in vitro* assays involving SrUGT76G1 such as Reb B and Reb M.

**Figure 3:**
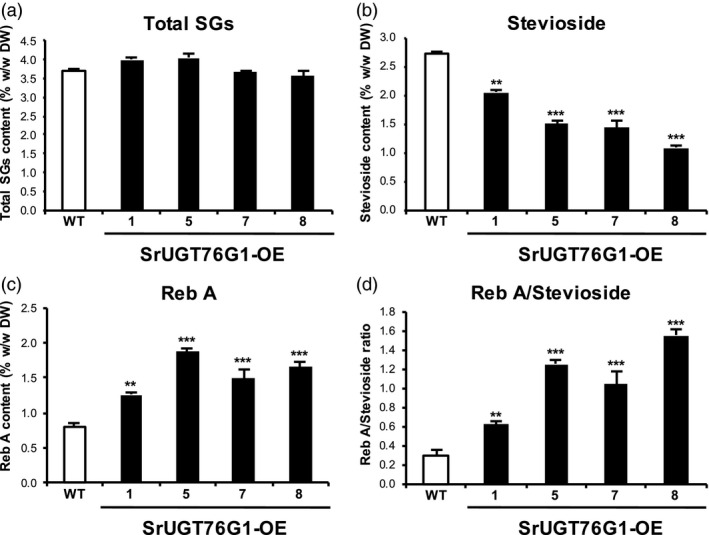
Analysis of steviol glycosides (SGs) content in SrUGT76G1‐OE lines. (a) The total concentration of SGs derived from the sum of the top four SGs (stevioside, Reb A, Reb C, dulcoside A). (b and c) Concentration of stevioside (b) and Reb A (c) from dried leaves of SrUGT76G1‐OE lines and wild‐type (WT). (d) Ratio of Reb A to stevioside in SrUGT76G1‐OE lines and WT. All SGs detected by HPLC were expressed as a percentage of their dry weight (% w/w DW) with mean ± SE. Statistical analysis was carried out using student's *t*‐test relative to WT plants (*n *=* *5, *P < 0.05, **P < 0.01, and ***P < 0.001).

For a more detailed study on the SGs content, we quantified the peaks of the top four most abundant SGs present in the leaves. By summing up four SGs, we could not see any significant difference in the total SGs content in the SrUGT76G1‐OE lines, which were between 3.56% and 4.04% (w/w dry weight; DW), compared to the 3.70% (w/w DW) in WT (Figure 3a). However, significant changes were observed in the individual SGs, especially for stevioside and Reb A, in the SrUGT76G1‐OE lines (Figure 3b and c). In contrast, neither the total SGs nor the proportion of individual SGs were significantly different in the internal control line #2 (Figure S3b–g). Compared to WT which has stevioside content of 2.71% (w/w DW), the transgenic lines showed content that were between 25% and 61% lower. For instance, the transgenic line with the lowest stevioside content, line #8, had concentration of only 1.07% (w/w DW). Even line #1 that possessed the highest stevioside content among the transgenic lines at 2.04% (w/w DW), was still 25% lower than that of WT (Figure 3b). It should be noted that the reduction of stevioside in transgenic lines correlated negatively with *SrUGT76G1* expression levels (Figure 1e and 3b).

On the other hand, Reb A content in the SrUGT76G1‐OE lines was significantly increased by up to 137.3% compared to WT (Figure 3c). In WT, the Reb A content was 0.79% (w/w DW), but in lines #1 and #5 that had the lowest and highest Reb A content, this was increased to 1.26% (w/w DW) and 1.87% (w/w DW) respectively (Figure 3c). To quantify the relative increase in Reb A to stevioside in the transgenic lines, we further calculated the ratio of Reb A to stevioside (Reb A/stevioside ratio) and observed a remarkable improvement in this ratio compared to WT. In WT, the Reb A/stevioside ratio was 0.30 and this increased to 0.62, 1.04, 1.25 and 1.55 in the SrUGT76G1‐OE lines #1, #7, #5 and #8 respectively (Figure 3d). That is, the Reb A/stevioside ratio was enhanced by about 207% to 517% in the SrUGT76G1‐OE lines. The higher Reb A/stevioside ratio was positively correlated with the transcript levels of *SrUGT76G1* in the SrUGT76G1‐OE lines (Figure 1e and 3d). Among the SrUGT76G1‐OE lines, line #8 had both the greatest Reb A/stevioside ratio and the highest *SrUGT76G1* expression levels, while line #1 showed the opposite (Figure 1e and 3d). These results demonstrate for the first time that SrUGT76G1 could indeed convert stevioside to Reb A *in planta* as well.

Other than changes in Reb A/stevioside ratio, the proportion of Reb C to dulcoside A was also affected. The dulcoside A concentration in the SrUGT76G1‐OE lines was between% 13.2% and 38.0% lower than that in the WT (Figure S4a). On the other hand, Reb C content was increased by between 17.2% and 37.8% in the transgenic lines compared to WT (Figure S4b). These results imply that SrUGT76G1 might be involved in the conversion of dulcoside A to Reb C in Stevia.

### Phenotypes of Stevia with SrUGT76G1 overexpression

**Figure 4:**
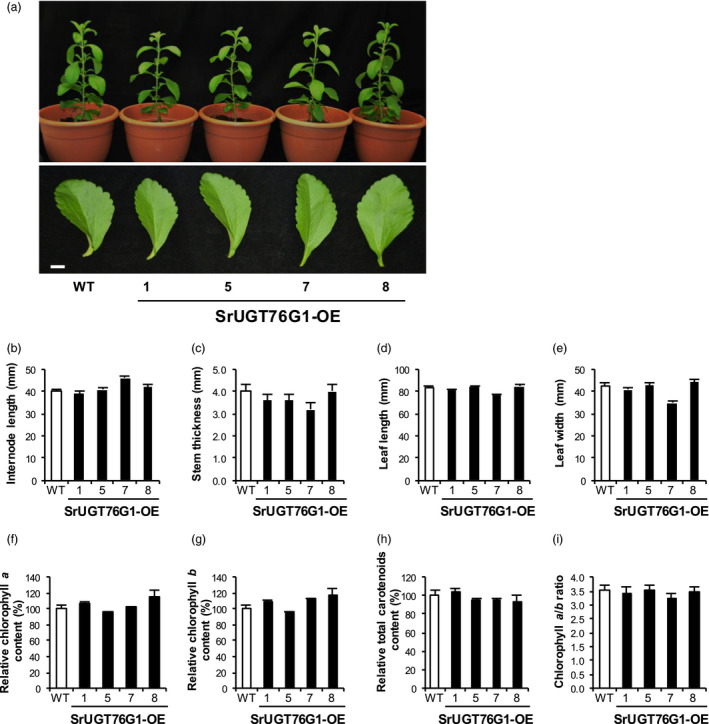
Phenotypic analysis of SrUGT76G1‐OE lines. (a) Upper panel, representative whole transgenic Stevia plants overexpressing *SrUGT76G1* (SrUGT76G1‐OE). Lower panel, leaf harvested from third node position of 2‐month old SrUGT76G1‐OE lines. (b and c) Average length (b) and thickness (c) of the third and fourth internodes from 2‐month old SrUGT76G1‐OE lines. (d and e) Average length (d) and width (e) of leaves from the third node. (f–h) Relative contents of chlorophyll *a* (f), chlorophyll *b* (g) and total carotenoids (h) in leaves from SrUGT76G1‐OE lines. (i) Ratio of chlorophyll *a* to *b*. All measurements were expressed as mean ± SE (*n *=* *5). WT, wild‐type. Scale bar = 10 mm.

Plants exhibit phenotypic changes such as dwarfism and reduced internode length under reduced GA content (Thomas and Sun, 2004). It has been reported that transient knockdown of the *SrUGT76G1* in Stevia led to an increase in GA levels so the overexpression of *SrUGT76G1* may have an opposite effect (Guleria and Yadav, 2013). Hence, we monitored the growth and development of the transgenic Stevia plants. Figure 4a shows that there were no obvious differences in morphology between the transgenic lines and WT. Internode length measurements made at 8 weeks after the transfer into the soil were comparable between the WT and SrUGT76G1‐OE lines at 40 mm and between 39 and 46 mm respectively (Figure 4b). In addition, the stem thickness and leaf size of the SrUGT76G1‐OE lines were also very similar to those of WT (Figure 4c–e).

We further quantified chlorophylls and total carotenoids content, which are essential metabolites that share some precursors with SGs biosynthesis (Rodríguez‐Concepción and Boronat, 2002). Similarly, the content of these metabolites in the Stevia with *SrUGT76G1* overexpression did not differ from WT (Figure 4f–h). Additionally, the chlorophyll *a*/*b* ratios were also comparable to that of WT indicating that the photosynthetic capacity of the transgenic lines was very similar to WT (Figure 4i). Therefore, other than changes in the Reb A/stevioside ratio, we could not find any other abnormalities in SrUGT76G1‐OE lines compared to WT Stevia plant.

### Expression pattern of other SGs pathway genes

**Figure 5:**
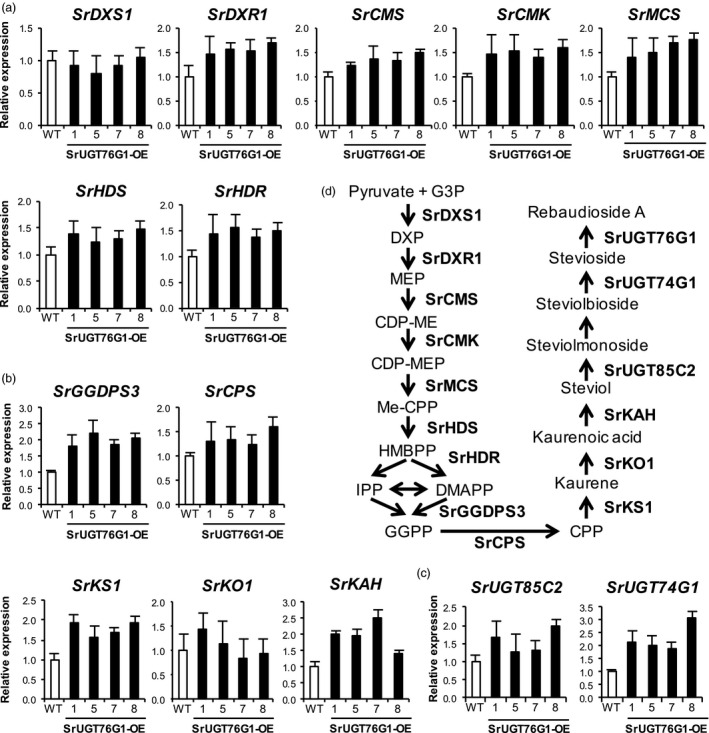
Transcript levels of genes in the SGs biosynthesis pathway in SrUGT76G1‐OE lines. (a–c) Transcript levels of genes involved in the methylerythritol phosphate (MEP) pathway (a), isoprenoid biosynthesis (b) and the glycosylation of steviol (c). All measurements were expressed as mean ± SE (*n *=* *3). WT, wild‐type; *SrDXS1, 1‐deoxy‐* *d* *‐xylulose 5‐phosphate synthase 1*;* SrDXR1*,* 1‐deoxy‐* *d* *‐xylulose 5‐phosphate reductoisomerase*;* SrCMS*,* 4‐(cytidine 5′ diphospho)‐2‐*C*‐methyl‐* *d* *‐erythritol synthase*;* SrCMK, 4‐(cytidine 5′ diphospho)‐2‐*C*‐methyl‐* *d* *‐erythritol kinase*;* SrMCS*,* 2‐*C*‐methyl‐* *d* *‐erythritol 2,4‐cyclodiphosphate synthase*;* SrHDS*,* (E)‐4‐hydroxy‐3‐methylbut‐2‐enyl diphosphate synthase*;* SrHDR*,* (E)‐4‐hydroxy‐3‐methylbut‐2‐enyl diphosphate reductase*;* SrGGDPS3*,* Geranylgeranyl diphosphate synthase 3*;* SrCPS*,* Copalyl pyrophosphate synthase*;* SrKS1*,* Kaurene synthase 1*;* SrKO1*,* Kaurene oxidase 1*;* SrKAH*,* Kaurenoic acid hydroxylase; SrUGT85C2, UDP‐glycosyltransferase 85C2; SrUGT74G1, UDP‐glycosyltransferase 74G1*. (d) Steps catalysed by each enzyme in the SGs biosynthesis pathway. G3P, Glyceraldehyde‐3‐phosphate; DXP, 1‐deoxy‐d‐xylulose 5‐phosphate; CDP‐ME, 4‐(cytidine 5′ diphospho)‐2‐C‐methyl‐d‐erythritol; CDP‐MEP, 4‐(cytidine 5′ diphospho)‐2‐C‐methyl‐d‐erythritol 2‐phosphate; ME‐cPP, 2‐C‐methyl‐d‐erythritol 2,4‐cyclopyrophosphate; HMBPP, (E)‐4‐hydroxy‐3‐methylbut‐2‐enyl pyrophosphate; IPP, isopentenyl pyrophosphate; DMAPP, dimethylallyl pyrophosphate; GGPP, Geranylgeranyl pyrophosphate; CPP, Copalyl pyrophosphate.

To investigate if the overexpression of *SrUGT76G1* somehow triggers a feedback loop that affects the expression of other genes in the SGs biosynthesis pathway, we measured the transcript levels of genes involved in the synthesis of the steviol precursor. Figure 5a and b shows that the expression of all gene except *SrDXS1* in the MEP pathway as well as the downstream genes for isoprenoid biosynthesis such as *SrGGDPS3*,* SrCPS*,* SrKS1*,* SrKO1* and *SrKAH,* were only slightly up‐regulated in the SrUGT76G1‐OE lines compared to WT, with all showing <3‐fold change. These results could possibly explain the minimal changes in total SGs content observed in the transgenic lines (Figure 3a). Moreover, the expression of *SrDXS1*, which has been suggested to be a rate‐limiting enzyme gene in the MEP pathway, was not changed in the SrUGT76G1‐OE lines (Estévez *et al*., 2001). The transcript abundance for two other SrUGTs, SrUGT85C2 and SrUGT74G1, was also slightly increased in the SrUGT76G1‐OE lines by up to around threefold (Figure 5c). However, this slight change in SrUGTs levels also did not affect the amount of rubusoside, which can be synthesized by the combined activities of SrUGT85C2 and SrUGT74G1 on steviol (Figure 2; Humphrey *et al*., 2006).

### An additional function of SrUGT76G1

In addition to the changes in Reb A/stevioside ratio, the Reb C/dulcoside A ratio in the SrUGT76G1‐OE lines was also affected (Figure S4b). SrUGT76G1 has so far been shown to be involved in 1,3‐glucosylations of C_13_‐ and C_19_‐positioned glucose of eight different SGs *in vitro* (Olsson *et al*., 2016). However, the potential conversion of dulcoside A to Reb C by the 1,3‐glucosylation activity of SrUGT76G1 has not yet been demonstrated.

**Figure 6:**
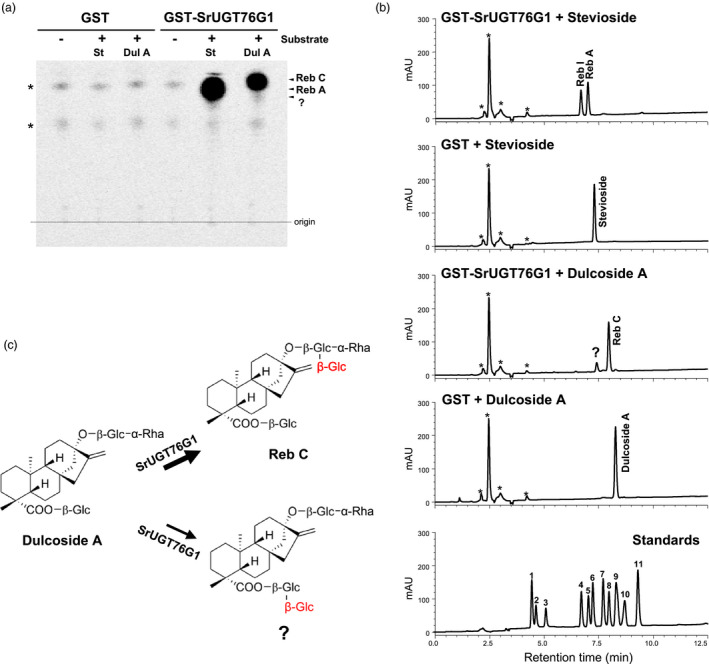
SrUGT76G1 activity assay using dulcoside A substrate. (a and b) Chromatograms from TLC (a) and HPLC (b) after reaction between dulcoside A and GST‐SrUGT76G1 or GST‐only. Standards, 11 SGs authentic standards. (c) Proposed schematic glucosylation reaction performed by SrUGT76G1 on dulcoside A. St, stevioside; Dul A, dulcoside A; Glc, glucose; Rha, rhamnose; 1, Reb E; 2, Reb D; 3, Reb M; 4, Reb I; 5, Reb A; 6, Stevioside; 7, Reb F; 8, Reb C; 9, Dulcoside A; 10, Rubusoside; 11, Reb B. mAU, milli‐Absorbance Units. Asterisks in (a) and (b) indicate nonspecific spots or peaks derived from *in vitro* assays.

To determine if SrUGT76G1 has an additional function for Reb C production from dulcoside A, we performed *in vitro* assays using recombinant SrUGT76G1 protein with UDP ‐glucose as the sugar donor and dulcoside A as the acceptor. Thin‐layer chromatography (TLC) analysis shows that the glutathione S‐transferase (GST)‐fused SrUGT76G1 (GST‐SrUGT76G1) recombinant protein, but not GST alone, was able to produce Reb C from dulcoside A in the reaction mixture (Figure 6a). In the positive control using stevioside as the acceptor, Reb A was obtained as expected (Figure 6a).

We further verified this reaction by HPLC analysis. In the positive control assay, Reb A was produced from the reaction mix containing GST‐SrUGT76G1 with stevioside (Figure 6b). An additional peak for Reb I, which can be converted from Reb A by GST‐SrUGT76G1, was detected as well (Figure 6b; Olsson *et al*., 2016). Most importantly, Reb C was detected in the reaction mixture with dulcoside A, confirming our TLC analysis. This result demonstrates that in addition to SGs acceptors reported so far, SrUGT76G1 also performs 1,3‐glucosylation on the C_13_‐positioned glucose on dulcoside A to form Reb C (Figure 6c). Moreover, it was confirmed *in planta* by the increased Reb C content and a concurrent decreased dulcoside A content observed in the SrUGT76G1‐OE lines (Figure S4). Interestingly, we detected another reaction product in our HPLC analysis with a retention time that does not correspond to any of our standards (Figure 6b). Since SrUGT76G1 catalyses 1,3‐glucosylation of C_13_‐ or C_19_‐positioned glucose of SG, we postulate that this novel peak is likely to be produced *in vitro* from the 1,3‐glucosylation of the C_19_‐positioned glucose on dulcoside A (Figure 6c). There was no peak obtained by the reaction of GST‐SrUGT76G1 without substrate (Figure S5).

## Discussion

The pleasant taste of Reb A and its relative abundance in Stevia has made it one of the most commercially valuable SGs that can be extracted from Stevia leaves. However, as its abundance is less than stevioside, which has a relatively stronger bitter aftertaste, it is desirable to generate new cultivars with higher Reb A levels. Here, we demonstrate that by overexpressing SrUGT76G1 in Stevia, we could generate new lines with higher Reb A/stevioside ratio.

SrUGT76G1 is known to convert stevioside to Reb A in *in vitro* assays (Figure 6a and b; Richman *et al*., 2005). When we overexpressed SrUGT76G1 in Stevia, we did not observe significant changes in the total SGs content compared to control, but the relative amount of individual SGs in each line changed. In WT, stevioside content was at 2.71% (w/w DW) but this was lowered to between 1.07% (w/w DW) and 2.04% (w/w DW) in the SrUGT76G1‐OE lines (Figure 3b). Conversely, we detected an increase in Reb A content to between 1.26% (w/w DW) and 1.87% (w/w DW) from the 0.79% (w/w DW) found in WT (Figure 3c). This translates to an increase in Reb A/stevioside ratio to between 0.62 and 1.55 in the overexpression lines from just 0.30 in the WT (Figure 3d). This change in ratio correlated positively with the transcript levels of SrUGT76G1 except for line #5, where the Reb A/stevioside ratio was much higher than in line #7 despite its lower expression levels of SrUGT76G1 (Figure 1e and 3d). We postulate that this was due to unexpected changes in translation rates or post‐translational regulations that may have conferred SrUGT76G1 with higher activity than usual at the protein level. When we further examined the transcript levels of other genes involved in the SGs biosynthesis pathway, the changes from WT were mostly less than threefold, which are much lower than the at least 14‐fold change in *SrUGT76G1* transcript (Figure 5). Thus, we believe that the overexpression of SrUGT76G1 had enhanced the conversion of stevioside to Reb A in the transgenic lines, making it an effective approach for increasing Reb A content in Stevia.

Other than a change in the Reb A/stevioside ratio, we also observed an increase in Reb C content relative to dulcoside A (Figure S4). It was previously suggested that Reb A and Reb C might be formed by the same or very closely linked enzyme because their proportions in the next generation are positively correlated (Brandle, 1999). Although it was discovered that SrUGT76G1 could convert stevioside to Reb A, the biosynthesis of Reb C remained unclear (Richman *et al*., 2005). However, in this study, Reb A and Reb C can be produced from the 1,3‐glucosylation on the C_13_‐positioned glucose of stevioside and dulcoside A respectively. By carrying out *in vitro* assays using purified recombinant SrUGT76G1 and dulcoside A, we detected Reb C as a product (Figure 6a and b). This confirms that Reb A and Reb C could certainly be synthesized by a common enzyme which we identify here to be SrUGT76G1. And the overexpression of SrUGT76G1 promoted the conversion of dulcoside A to Reb C in Stevia as well.

Plant UGTs have the potential to accept a broad range of substrates but show regiospecificity (Hansen *et al*., 2003). Earlier *in vitro* assays with SrUGT76G1 showed that it could carry out 1,3‐glucosylation at the C_13_‐ or C_19_‐ positioned glucose of several substrates including, stevioside, steviobioside, rubusoside, Reb A, Reb D, Reb E and Reb G (Olsson *et al*., 2016). Our identification of dulcoside A as a substrate of SrUGT76G1 further adds to this list. However, these *in vitro* conversions did not all translate into *in vivo* observations in the Stevia with *SrUGT76G1* overexpression. In particular, although Reb A was converted by SrUGT76G1 into Reb I *in vitro* (Figure 6b; Olsson *et al*., 2016), Reb I was not detectable in the SrUGT76G1‐OE lines despite their elevated Reb A levels (Figure 2). Furthermore, we did not detect any significant increase in the Reb B content in the SrUGT76G1‐OE lines even though steviobioside, which is a precursor to stevioside, could be converted by SrUGT76G1 into Reb B *in vitro* (Olsson *et al*., 2016). This suggests that on top of regiospecificity, other factors such as compartmentalization and enzyme affinity can influence the substrate specificity of SrUGT76G1 *in vivo*. However, such differences between *in vitro* and *in vivo* function of UGTs are not limited to SrUGT76G1 in Stevia. For example, AtUGT73C6 was found to only have flavonol‐3‐*O*‐glycoside‐7‐*O*‐glucosyltransferase activity in Arabidopsis but it could also glucosylate isoflavones and flavanoid aglycones *in vitro* (Bowles *et al*., 2005; Jones *et al*., 2003). However, the possibility that the concentrations of several substrates for SrUGT76G1 such as rubusoside, Reb E and Reb G are present only in minute amounts in Stevia leaves, resulting in their products being undetected, should not be excluded.

Generally, dried Stevia leaves are reported to contain between 4% and 20% of SGs (Lemus‐Mondaca *et al*., 2012). Our Stevia has SGs content of around 4%. This low content of SGs may be due to environmental factors as we have grown them under local climate which is warmer and shorter in day length compared to its native environment (Lemus‐Mondaca *et al*., 2012). Nevertheless, with changes in the proportion of the major SGs in the SrUGT76G1‐OE lines, the total extract from the leaves is expected to have improved taste. By considering Reb A and stevioside that together make up more than 90% of all SGs in the leaves, Reb A, which is perceived to be sweeter than stevioside, is increased by up to 137.3% in contrast to the decrease of up to 61% in stevioside content in the SrUGT76G1‐OE lines. Even among the two other major SGs that are considered less desirable due to their strong bitter taste, Reb C, which is slightly sweeter and less bitter, had an increase of up to 38% in content compared to the similar extent of decrease in dulcoside A. Therefore, the overexpression of SrUGT76G1 in Stevia could efficiently enhance the taste of Stevia leaf extracts.

In conclusion, the overexpression of SrUGT76G1 can alter the SGs profile in Stevia, increasing the ratio of Reb A to stevioside and to a smaller extent the ratio of Reb C to dulcoside A. Moreover, other than converting stevioside to Reb A, SrUGT76G1 is also shown to carry out 1,3‐glucosylation on dulcoside A to produce Reb C *in vitro*. Since both these conversions led to an increase in the proportion of the more pleasant tasting SG within each pair, *SrUGT76G1* overexpression in the Stevia plant serves as an effective way to generate new varieties with chemotypes that are more commercially valuable.

## Experimental procedures

### Stevia transformation

The full‐length ORF of SrUGT76G1 (Accession number, AY345974; Richman *et al*., 2005) that was PCR‐amplified from the cDNA of Stevia leaves using primers listed in Table S1 was cloned into the pK7WG2D vector using GATEWAY technology (Invitrogen, Carlsbad, CA). After confirmation by sequencing, the expression vector was transformed into the *Agrobacterium* strain AGL2. Transformation of Stevia using this *Agrobacterium* was well described (Zheng *et al*., 2018). Briefly, the leaf explants were co‐cultivated with *Agrobacterium* on co‐cultivation media (0.25 mg/L 2,4‐dichlorophenoxyacetic acid (2,4‐D) + 100 μm acetosyringone) for 3 days and transferred onto callus induction media (1 mg/L 6‐benzylaminopurine (BA) + 0.5 mg/L 3‐indoleacetic acid (IAA) + 125 mg/L cefotaxime + 50 mg/L kanamycin) after washing. For visual selection of transformants, we used GFP gene fused to an endoplasmic reticulum targeting signal (*EgfpER*) under the control of rolD promoter that is highly active especially in roots and calli (Figure 1a; Karimi *et al*., 2007). After 3–4 weeks of incubation on callus induction media containing kanamycin, transformed calli that emitted GFP signals under a fluorescent microscope were further transferred onto shoot induction media (2 mg/L BA + 0.25 mg/L IAA + 125 mg/L cefotaxime + 50 mg/L kanamycin) and subcultured every 3–4 weeks. The explants were incubated at 25 °C in the dark throughout. Regenerated shoots with GFP signals were then transferred onto rooting media (0.5 mg/L IAA + 125 mg/L cefotaxime) under long day (LD) condition (16 h Light/8 h Dark). Fully developed transgenic plants were propagated *in vitro* by cutting method and transferred onto soil after roots developed. For hardening, plants were placed in a plant growth chamber at 25 °C with exposure to LD condition and covered with a transparent plastic dome. Subsequently, plants were shifted to the greenhouse and subjected to the local climate conditions.

### Verification of transgenic Stevia plants by genomic PCR and Southern blot analysis

Cetyltrimethylammonium bromide (CTAB)‐based extraction method was used to extract genomic DNA (gDNA) from Stevia leaves (Rogers and Bendich, 1989).

For genomic PCR, approximately 100 ng of gDNAs was added to a PCR reaction mix containing forward and reverse primers specific to the CaMV 35S promoter and to the 3′ end of *SrUGT76G1* respectively (Table S1).

Southern blot analysis was carried out using a digoxigenin (DIG)‐labelled probe specific to the full length of the CaMV 35S promoter. gDNAs extracted from the SrUGT76G1‐OE lines were digested with *Hin*dIII and resolved on a 0.8% agarose gel together with the DIG‐labelled DNA molecular weight marker II (Roche, Mannheim, Germany). The agarose gel was then treated for the transfer of fragmented gDNAs onto a positively charged nylon membrane (Hybond‐N+) as mentioned previously (Zheng *et al*., 2018). Following DIG‐based Southern blot hybridization (Roche), chemiluminescence from the membrane was detected using the ChemiDoc Touch Imaging System (Bio‐Rad, Hercules, CA).

### Expression analysis by quantitative real‐time PCR (qRT‐PCR)

Total RNA was extracted from homogenized Stevia leaves using TRIzol reagent (Invitrogen) and contamination from DNA was removed after treatment with deoxyribonuclease I (DNase I; Roche). For cDNA synthesis, 1 μg of total RNA was used with M‐MLV Superscript II (Promega, Madison, WI). To determine the transcript abundance of *SrUGT76G1* and all other genes in the SGs biosynthesis pathway, qRT‐PCR was performed using SYBR Premix Ex Taq II (Takara, Shiga, Japan) and quantified on Applied Biosystems (USA) 7900HT fast real‐time PCR system. Primers used are listed in Table S1. Primer specificity was verified by sequencing of product from regular PCR and melting curve analysis. The abundance of Stevia *actin* transcript was used as an internal control for normalization.

### Steviol glycosides content analysis by High‐Performance Liquid Chromatography

Stevia leaves for SGs content analysis were harvested from the sixth node of plants grown in the greenhouse for 3 weeks. After drying the leaves overnight in a 60 °C oven, the samples were ground and 30 mg of the powdered leaves were extracted using 3 mL of water twice in an ultrasonic bath maintained at 50 °C for 20 min. The extracts were centrifuged at 1800 × g for 15 min. One microlitre of supernatant filtered through a 0.45 μm filter was loaded onto a solid phase extraction (SPE) column C2 (Agilent, Santa Clara, CA) and washed with acetonitrile:water (20:80, v/v) before elution in 1 mL of methanol:acetonitrile (50:50, v/v). To analyse SGs content, 5 μL of the eluted sample was applied on a Shimadzu Nexera ×2 ultra‐high performance liquid chromatography (UHPLC) fitted with a Shim‐pack VP‐ODS column (250 × 4.6 mm, i.d. 5 μm) and detected by a photodiode array detector (SPD‐M30A with high sensitivity cell). The elution was performed over 24 min with a 30%–80% acetonitrile gradient at a flow rate of 1.0 mL/min. Column oven was maintained at 40 °C. Chromatogram detected at a wavelength of 210 nm was used for SGs identification and quantification. Peak assignment was based on comparison with elution profile of known standards (ChromaDex,Irvine, CA) and the concentration of each SG was determined from the standard curves of the respective SGs.

### Chlorophylls and total carotenoids analysis

For the measurement of chlorophylls and total carotenoid content, Stevia leaves were harvested from the fourth and fifth nodes of plants that were grown in the greenhouse for 3 weeks and frozen in liquid nitrogen. After homogenization, 200 mg of the powdered leaves was extracted twice with 2 mL of 100% methanol at room temperature for 1 h with constant shaking in the dark. The extracts were pooled and diluted fivefolds before analysis on an Infinite M2000 microplate reader (Tecan, Männedorf, Switzerland). Absorbance values at three different wavelengths, 666 nm, 653 nm and 470 nm, were used to calculate the relative amount of chlorophyll *a*, chlorophyll *b* and total carotenoids present in the leaves based on previously reported formula (Lichtenthaler and Wellburn, 1983).

### Expression of recombinant SrUGT76G1 and UDP‐glucosyltransferase activity assay

The full‐length cDNA of *SrUGT76G1* was cloned into pDEST15 to obtain a GST‐tagged fusion protein where the GST is fused to the N‐terminus. The resulting expression vector was transformed into *E. Coli* BL21 (DE3)‐derived Rosetta strain (Novagen, Darmstadt, Germany). GST‐tagged SrUGT76G1 recombinant protein was purified by glutathione agarose beads (ThermoFisher Scientific, Waltham, MA). About 1 μg of recombinant protein was used for enzyme assay with 50 μm of the substrate (dulcoside A or Reb A) in an assay buffer (50 mm HEPES, pH 7.5, 3 mm MgCl_2_, 10 μg/mL Bovine Serum Albumin). To initiate the reaction, a 1 mm UDP‐glucose mixture (997.5 μm UDP‐glucose and 2.25 μm UDP‐[^14^C]‐glucose, 2.78 kBq, Amersham Biosciences, Buckinghamshire, UK) was added. *In vitro* glucosyltransferase activity assays were performed as described by Richman *et al*. (2005). The assay was carried out at 30 °C for 2 h and extracted twice with 100 μL of water‐saturated 1‐butanol. Pooled fractions were dried in a vacuum centrifuge and resuspended in 10 μL water‐saturated 1‐butanol for thin‐layer chromatography (TLC) analysis. The TLC was performed with 10 μL of reaction products using chloroform: methanol: water (15:10:2 v/v/v) as the mobile phase on a silica gel‐coated TLC plate (Fluka, Buchs, Switzerland) in a mobile phase saturated glass chamber. SG standards were run under the same condition. After air‐drying, the image on the TLC plate was captured on a storage phosphor screen in a phosphorimager cassette (Bio‐Rad) for 2–3 d and visualized on a Typhoon 9200 imager (Amersham Biosciences).

### HPLC analysis of *in vitro* glucosyltransferase activity assay mixture

*In vitro* glucosyltransferase activity assay was performed as indicated in the TLC analysis, but 5 mm of UDP‐glucose was used without UDP‐[^14^C]‐glucose and the incubation time was increased to 16 h. Samples were extracted three times with water‐saturated 1‐butanol and dried completely in a vacuum centrifuge. Dried samples were dissolved in MeOH for UHPLC analysis in accordance with the method mentioned for SGs content analysis.

## Conflict of interest

The authors declare that they have no conflict of interest.

## Supporting information


**Figure S1** Glucosylation pathway for the biosynthesis of rebaudioside A.
**Figure S2** Original image of Southern blot analysis shown in Figure 1d.
**Figure S3** Steviol glycosides (SGs) content in SrUGT76G1‐OE #2.
**Figure S4** Dulcoside A and Reb C content in SrUGT76G1‐OE lines.
**Figure S5** *In vitro* assay of GST‐protein activity.

**Table S1** List of primers used in study.
