# Supplementary material for: Overexpression of SrUGT76G1 in Stevia alters major steviol glycosides composition towards improved quality
Source: Plant Biotechnol J. 2018 Dec 19;17(6):1037–47. doi: 10.1111/pbi.13035 (PMC6523589; doi:10.1111/pbi.13035)
Supplement: Supplementary file 1 — Figure S1 Glucosylation pathway for the biosynthesis of rebaudioside A. Figure S2 Original image of Southern blot analysis shown in Figure 1d. Figure S3 Steviol glycosides (SGs) content in SrUGT76G1‐OE #2. Figure S4 Dulcoside A and Reb C content in SrUGT76G1‐OE lines. Figure S5 In vitro assay of GST‐protein activity. [file PBI-17-1037-s001.pdf]

Supporting Figure S1

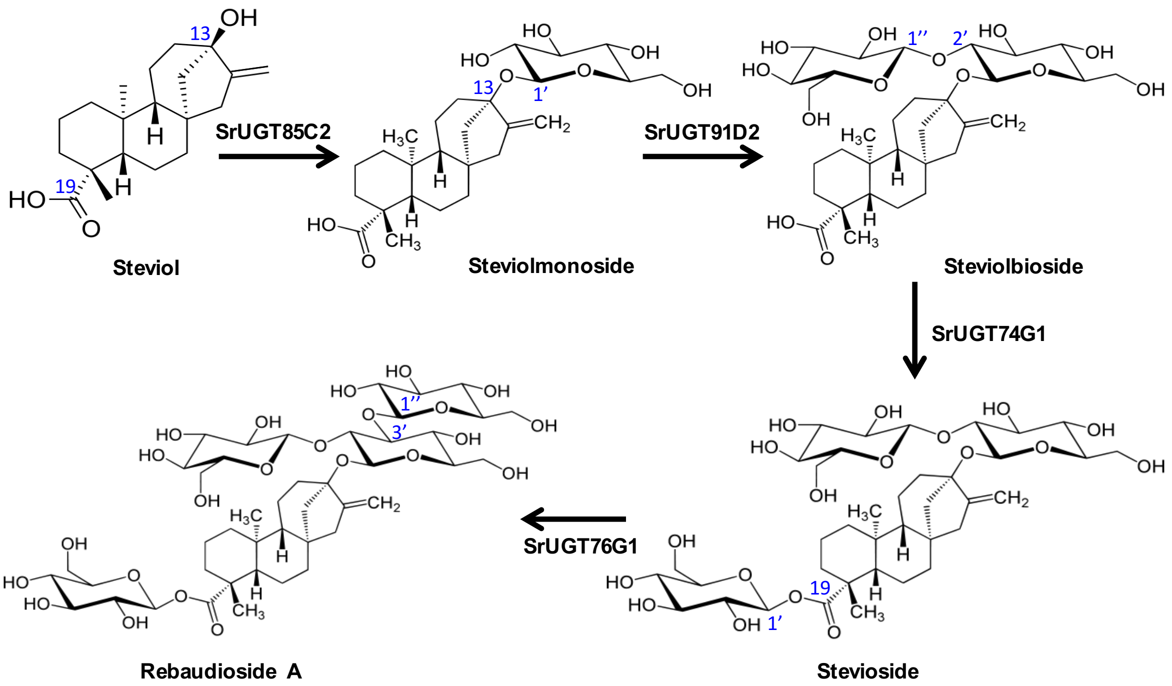

Supporting Figure S1 Glucosylation pathway for the biosynthesis of Rebaudioside A.
Steviol is first converted to steviolmonoside by SrUGT85C2 that adds a glucose moiety at the C_{13} hydroxyl position (Richman *et al.*, 2005; Humphrey *et al.*, 2006). SrUGT91D2 has been suggested to then carry out a 1,2-β-D-glucosylation on the glucose of steviolmonoside to form steviolbioside (Olsson *et al.*, 2016). Subsequently, SrUGT74G1 glucosylates steviolbioside on the C_{19} carboxyl group to produce stevioside (Richman *et al.*, 2005; Humphrey *et al.*, 2006). Finally, Rebaudioside A is formed from the addition of another glucose moiety with a 1,3-β-D-glucosidic linkage on stevioside by SrUGT76G1 (Richman *et al.*, 2005; Olsson *et al.*, 2016).

Supporting Figure S2

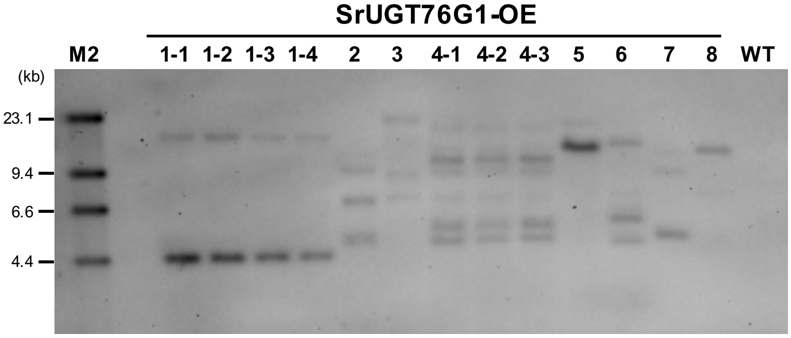

Supporting Figure S2 Original image of Southern blot analysis shown in Figure 1d.
4 individuals from SrUGT76G1-overexpression line #1 and 3 individuals from line #4 were loaded on this blot.

Supporting Figure S3

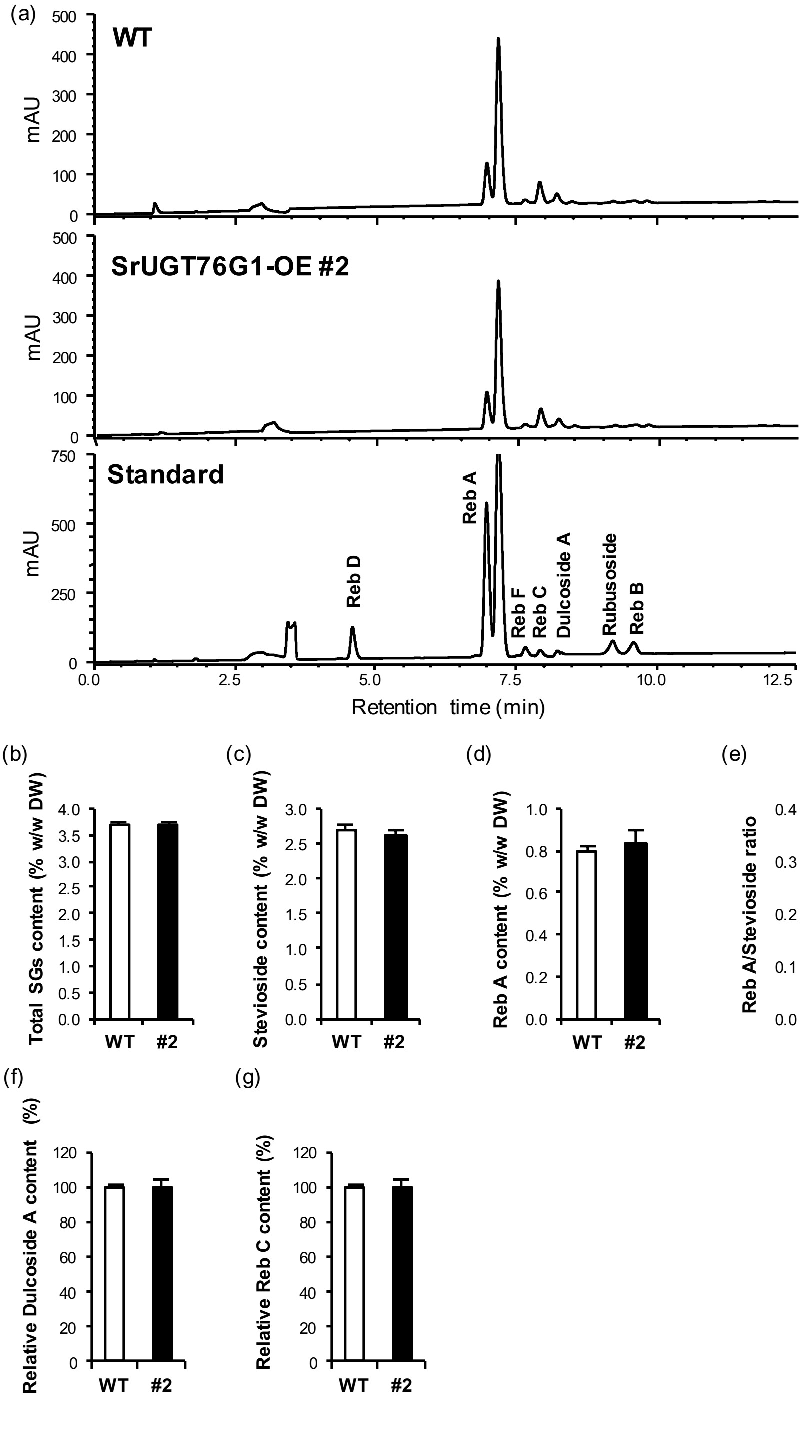

Supporting Figure S3 Steviol glycosides (SGs) content in SrUGT76G1-OE #2.
(a) HPLC chromatogram of SGs extracted from dried leaves of SrUGT76G1-OE line #2 and wild-type (WT). (b) The total concentration of SGs derived from the sum of the top four SGs (stevioside, Reb A, Reb C, dulcoside A). (c-e) Stevioside content (c), Reb A content (d) and the ratio of Reb A to steviode (e) detected in the leaves. (f and g) Relative Dulcoside A (f) and relative Reb C (g) content when compared to WT. Standard errors are represented by the error bars. Statistical analysis are carried out using student's *t*-test relative to WT plants (n=5, *$p<0.05$).

Supporting Figure S4

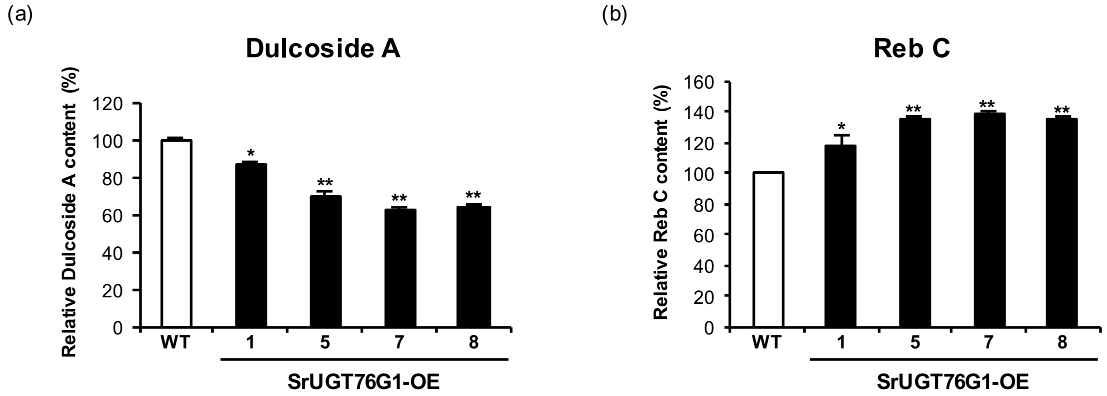

Supporting Figure S4 Dulcoside A and Reb C content in SrUGT76G1-OE lines.
(a and b) Dulcoside A (a) and Reb C (b) content in each line relative to their content in WT. All SGs were detected using HPLC. Standard errors are represented by the error bars. Statistical analysis are carried out using student's *t*-test relative to WT plants (n=5, *p<0.05, **p<0.01)

Supporting Figure S5

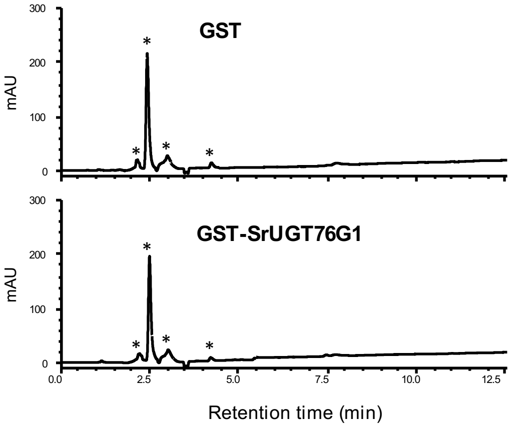

Supporting Figure S5 *In vitro* assay of GST-protein activity.
HPLC chromatograms of products from assay containing GST or GST-SrUGT76G1 only without substrate as a negative control. mAU, milli-Absorbance Units. The asterisks indicate nonspecific spots or peaks derived from *in vitro* assays.
